# CircMRE11A_013 binds to UBXN1 and integrates ATM activation enhancing lens epithelial cells senescence in age-related cataract

**DOI:** 10.18632/aging.202470

**Published:** 2021-01-28

**Authors:** Junliang Liu, Jinling Zhang, Guowei Zhang, Tianqiu Zhou, Xi Zou, Huaijin Guan, Yong Wang

**Affiliations:** 1 Eye Institute, Affiliated Hospital of Nantong University, Nantong, Jiangsu, China; 2 Department of Ophthalmology, The Third People’s Hospital of Changzhou, Jiangsu, China

**Keywords:** ultraviolet B, circular RNA, UBXN1, ATM activation, age-related cataract

## Abstract

Ultraviolet B (UVB) irradiation could trigger DNA double-strand breaks (DDSBs) and senescence in lens epithelial cells (LECs), thus inducing age-related cortical cataract (ARCC) formation. Cell-cycle irreversible arrest induced by DDSBs depended on excessive activation of ataxia-telangiectasia mutated kinase (ATM). We studied the up-regulated circular RNA circMRE11A_013 (circMRE11A) in LECs of ARCC and SRA01/04 cell lines under UVB exposure. *In vitro*, knockdown of circMRE11A in SRA01/04 cell lines enhanced cell viability and cell cycle, while over-expression of circMRE11A exhibited an opposite trend. Additionally, circMRE11A could bind to UBX domain-containing protein 1 (UBXN1), which might enhance excessive activation of ATM and initiate ATM/p53/p21 signaling pathway causing LECs cell-cycle arrest and senescence. *In vivo*, recombinant adeno-associated virus vectors (rAAV-2) virions of circMRE11A (circMRE11A-AAV2) was injected to Institute of Cancer Research mouse vitreous cavity. The circMRE11A-AAV2 could express in mouse lens at 4 weeks. The LECs aging and opacity lens were observed at 8 weeks after the injection. Together, our findings reveal a previously unidentified role of circMRE11A interacting with UBXN1 in enhancing ATM activity and inhibiting LECs cell-cycle in ARCC formation. The findings might give us a better understanding of ARC pathology and provide a novel and more effective therapeutic approaches for ARC treatment.

## INTRODUCTION

Age-related cataract (ARC) is a primary cause of blindness all around the world [1]. There are three types of ARC including cortical, nuclear and posterior subcapsular [2]. With the growth of aging population, visual impairment caused by ARC will rise [3]. However, the precise etiology and pathogenesis of ARC is not completely clear. Ultraviolet B (UVB) irradiation induced DNA damage is one of the most important environmental risk factors for ARC formation [4, 5]. Studies have demonstrated that UVB irradiation may directly cause DNA double-strand break (DDSBs) in the lens epithelial cells (LECs). It could trigger LECs senescence in ARC formation, especially in age-related cortical cataract (ARCC) [6–8]. However, the pathogenesis of ARCC formation due to UVB-induced DDSBs is not well understood.

Cellular senescence is a cell fate that entails essentially irreversible replicative arrest [9]. Cells could become pre-senescent when exposure to UVB. The replicative potential of DNA and cell cycle could become be restrained [10]. DNA oxidative damage repair and rapid DNA damage response (DDR) are required to preserve genome integrity [11]. ATM/p53/p21 signaling pathway is one of the major pathways to inhibit cell cycle due to DNA damage [9, 12, 13]. ATM, ataxia-telangiectasia mutated kinase, has a vital role in the maintenance of genome integrity, cell cycle arrest and controls the DNA repair network [14–16]. Mutations of the ATM gene often lead to gene instability and defect of DDR. Excessive activation of the ATM extensively contributes to multiple age-related diseases [17]. Earlier studies have demonstrated that the *atm* ^-/-^ mouse quickly died, while the *atm* ^+/-^ mouse showed radio sensitivity and was susceptible to cataract formation [14, 18, 19]. Our previous research only focused on the mRNA or proteins expression level of ATM in ARC [20]. However, evidence shows that unmodified proteins such as ATM have no biological activity *in* *vivo* [17]. Excessive activation of ATM is upon genotoxic stress, such as UVB, which might cause LECs senescence in ARCC, but the mechanism is not clear.

In the regular of the process of aging, noncoding RNAs play the critical roles [21]. Circular RNAs (circRNAs) are a class of noncoding RNAs characterized by the presence of covalently linked ends produced in a noncanonical splicing event called “back-splicing” [22]. CircRNAs are raising the intriguing possibilities of functional roles in cell senescence due to their circular structural features, resistant toward RNase R and specific expression patterns [23, 24]. Growing evidence supports that circRNAs participated in post-translational modifications (PTMs) of proteins is as competitive combination microRNAs (miRNAs) or scaffold of proteins [25]. The most common PTMs include phosphorylation, acetylation, glycosylation, ubiquitination, acetylation and hydroxylation [26, 27]. The ubiquitin modified of proteins caused the exposure of protein activation sites, such as the exposure of protein phosphorylation sites, which could trigger downstream signaling pathway [27]. However, few studies have provided information on circRNAs involved in PTMs of proteins during ARC formation.

Our previous studies have found that DDSBs in LECs of ARCC were obviously severer compared with controls. DNA oxidative damage repair genes played the critical roles in progression of ARC formation [7, 28]. *MRE11A* gene, a DNA oxidative damage repair gene, was down-expression in LECs of ARC patients compared with controls [29]. The change of expression may influence LECs vitality and lens transparent [28, 30]. The MRE11A also is an essential protein for ATM recruitment and activation to initiate the DNA repair damage pathway [31]. Hsa_circMRE11A_013 (http://www.circbank.cn/) is driving from *MRE11A,* containing 307 bp. We have identified it as circMRE11A. CircMRE11A may participate in the progress of LECs viability and proliferation as well as cell cycle control. Additionally, we found that circMRE11A might integrate activation of ATM (ATM-S1981p) in LECs senescence through binding to proteins UBX domain-containing protein 1 (UBXN1, also known as stress activated protein kinase substrate 1, SAKS1). *In vivo*, recombinant adeno associated virus (rAAV) vectors rAAV-2 virions of circMRE11A(circMRE11A-AAV2) could pass through the capsule tissues of lens and express in the lens of mouse after intravitreal injection at 4 weeks. Aging mouse LECs is increasing and the lens of mouse appear opacity at 8 weeks after the injection. In current study, as the scaffold of UBXN1, circMRE11A could induce excessive activation of ATM enhancing LECs senescence through ATM/p53/p21 signaling pathway. The finding might provide a new effective therapeutic molecular target.

## RESULTS

### Identification of circMRE11A in LECs of ARCC and SRA01/04 cell lines

**Figure 1:**
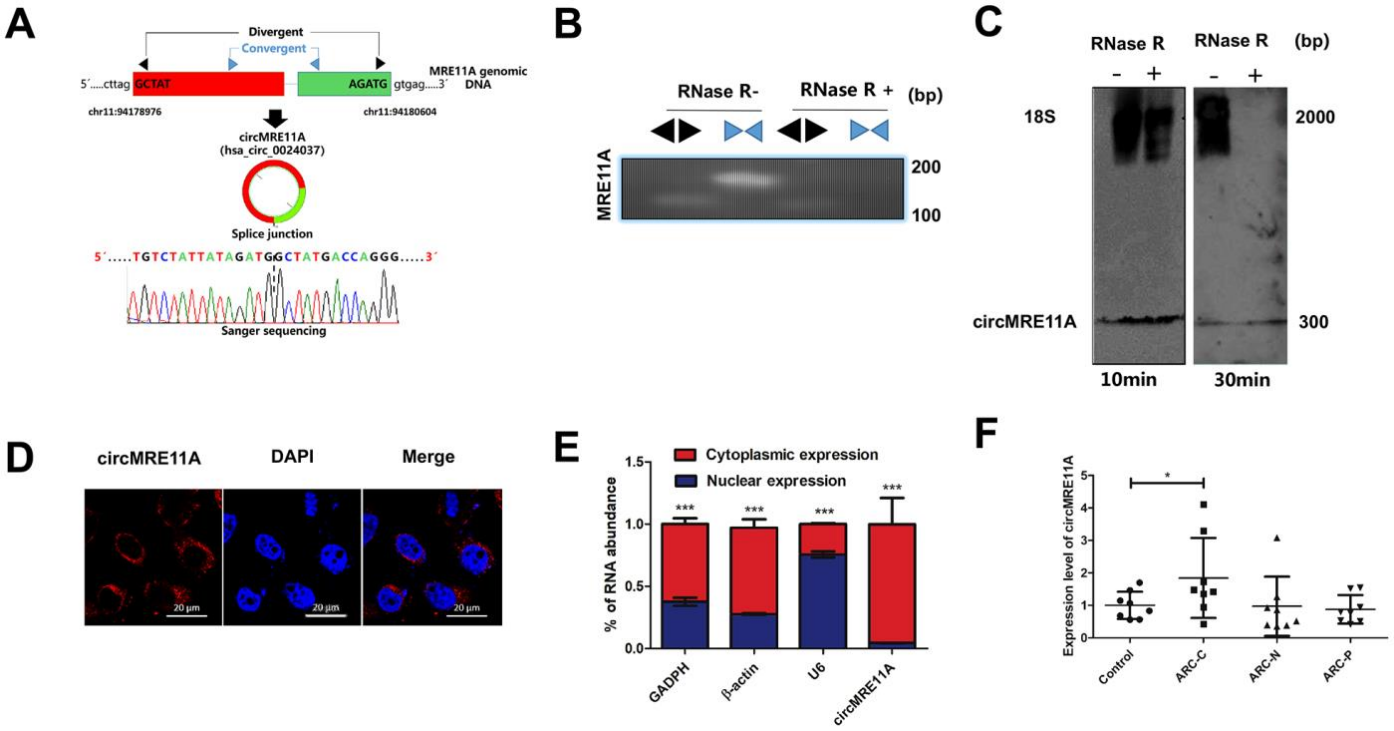
**Identification of circMRE11A, a circular RNA implicated in LECs of ARC and SRA01/04 cell lines, circMRE11A was up-regulated in ARCC compared with controls.** (**A**). Schematic illustration showed that the genomic location of circMRE11A generated from its host gene, validating by Sanger sequencing. Sanger sequencing analysis showed head-to-tail splicing junction of circMRE11A. (**B**). RT-PCR or PCR assay indicated the detection of circMRE11A using divergent or convergent primers from cDNA or genomic DNA (gDNA) of LECs with and without RNase R treatment (RNase R– / +). (**C**). Northern blot using a junction-specific the Bioth-labeled probe indicated the endogenous existence of circMRE11A treated with and without RNase R (2 U/μg) in the cell lines at 10 min or 30 min. 18S was used as a specific probe for the internal control. (**D**). RNA-FISH assay showed the cytoplasmic and nuclear localization of circMRE11A in SRA04/01 cells using a junction specific probe (red), with the nuclei staining with DAPI (blue). Scale bar: 20 μm. (**E**). qRT-PCR indicated the distribution of circMRE11A, GAPDH, β-actin and U6 in the cytoplasmic and nuclear fractions of SRA04/01 cells. U6 was treated as a nuclear control while GAPDH or β-actin was a cytoplasmic control, ***p < 0.001. (**F**). qTR-PCR assays determined circMRE11A levels in LECs of ARCs (controls (n=10), ARC-C(n=10), ARC-N (n=10), ARC-P(n=10)). GAPDH was an internal control. *p < 0.05.

The circMRE11A is derived from Exon 6 and Exon 7 of the parental transcript. Sanger sequencing confirmed that the head-to-tail splice junction was identical to the reported sequence in circBank (http://www.circbank.cn/) (Figure 1A). Indeed, total RNAs were digested with RNase R which could selectively degrades linear RNA. Next the head-to-tail splicing of endogenous circMRE11A was assayed by RT-qPCR with convergent and divergent primers, consistent with the circular form. The PCR product was amplified by the primers for circMRE11A but not *MRE11A* mRNA (Figure 1B). We further ruled out possibilities that the observed head-to-tail splicing was produced by trans-splicing, genomic rearrangements or even PCR artifacts by Northern Blot. Circularization of the expressed circMRE11A was confirmed by treating the samples with RNase R to digest linear 18 S mRNA but not circMRE11A. We confirmed that circMRE11A resolved at 307 nt consistent with the hsa_circ_000237 annotation (Figure 1C). The circRNA mainly expressed in the cytoplasm of LECs (Figure 1D) consistent with RNA separation of nucleus and cytoplasm (Figure 1E). We further found circMRE11A expression was significantly higher in ARCC group than that in controls (Figure 1F).

### Comparing with controls, LECs senescence increased in ARCC

**Figure 2:**
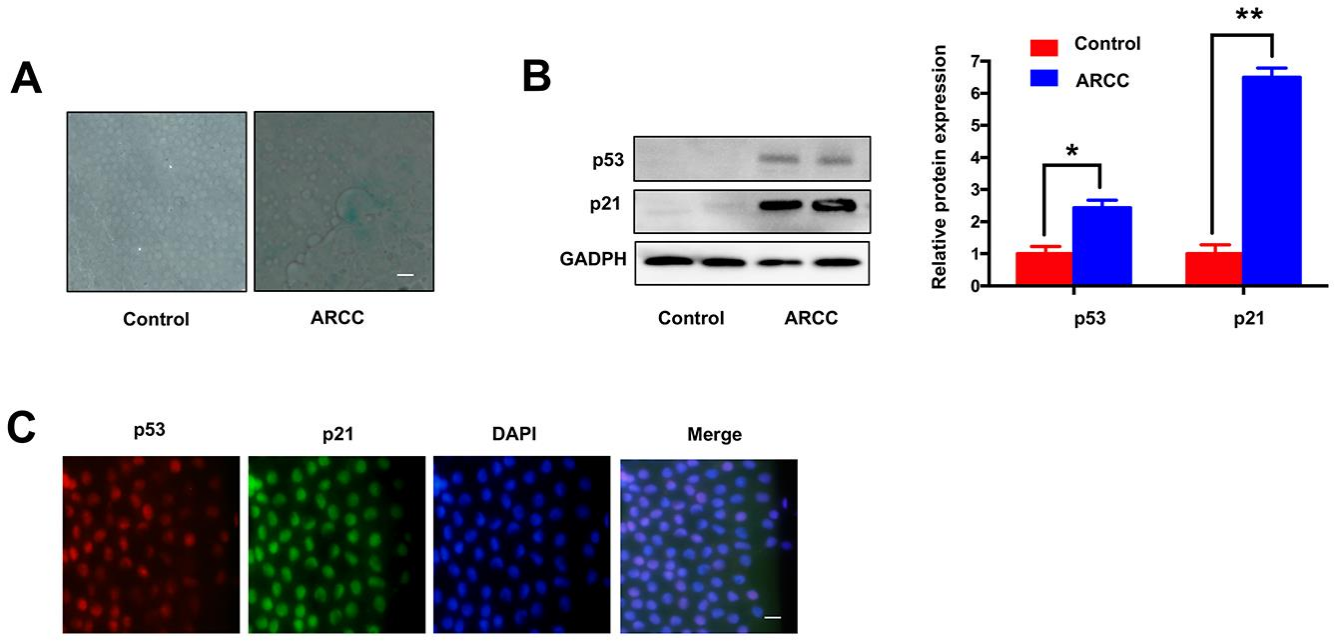
**Comparing with controls, LECs aging was increasing in ARCC.** (**A**). The number of SA-β-gal-positive cells increased in LECs of ARCC compared with controls. The senescence of LECs in ARCC was significantly increasing. Scale bar: 50μm. (**B**). The expression levels of proteins in ARCC and controls were detected by western blot, expression of aging related protein p53 and p21 were higher than controls. **p <0.01. (**C**). Immunofluorescence showed that p53 and p21 were co-located in the nucleus, while the upstream protein of ATM was located in the cytoplasm in ARCC. with the nuclei staining with DAPI (blue). Scale bar: 50 μm.

Consistent with the others described [8], there are increasing number of SA-β-gal-positive cells in LECs of ARCC. The phenomenon showed aging of LECs increased significantly when compared with controls (Figure 2A). Contrary to the results (Figure 1F), the expression of *MRE11A* mRNA down-regulated in LECs of ARCC. Western Blot showed the expression of aging related protein p53 and p21 in ARCC LECs were obviously higher than controls (Figure 2B). Moreover, immunofluorescence showed that p53 and p21 were co-located in the nucleus (Figure 2C). These results suggested that the senescent of LECs was an important pathophysiological process of ARCC and up-regulation of circMRE11A might link to the process.

### LECs senescence is raising under UVB-inducing oxidative damage *in vitro*

**Figure 3:**
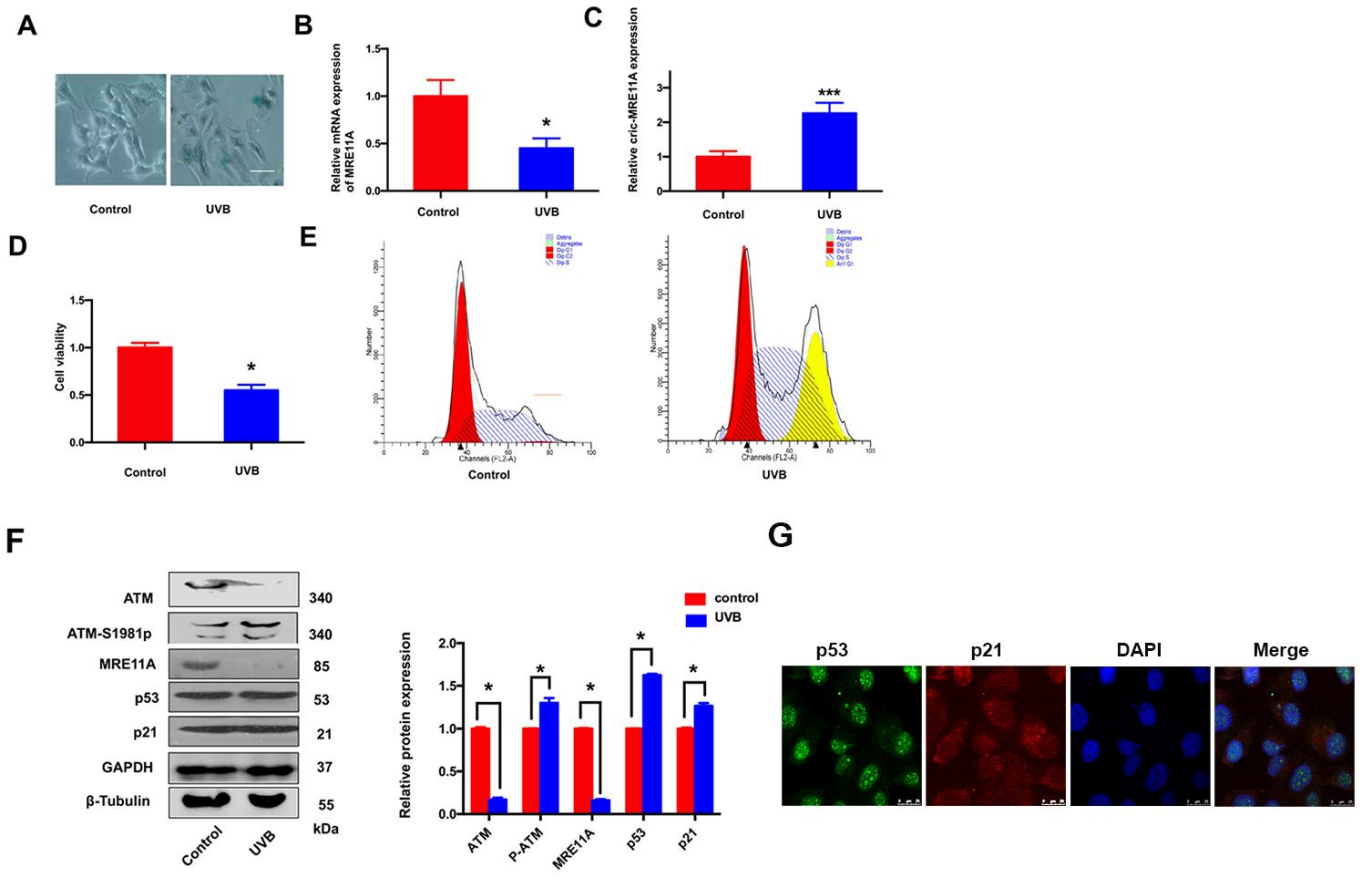
**LECs senescence was raising under UVB-inducing oxidative damage *in vitro*.** (**A**). The number of SA-β-gal-positive SRA01/04 cells exposed under UVB were increased than controls. Scale bar: 25μm. (**B**). When UVB induced cells aging, the expression level of MRE11A mRNA was down-regulation. *p<0.05. (**C**). The expression level of circMRE11A was up-regulation compared with controls. ***p<0.001. (**D**). CCK-8 showed that the cell viability in UVB group was decreased. *p<0.05. (**E**). Flow cytometry demonstrated that cell cycle was inhibited in G1/S in UVB group. (**F**). Western Blot showed that the expression of ATM or MRE11A proteins were decreased, while the expression level of ATM-S1981p was increasing in UVB group compared with controls. Meanwhile, the relative increase of p53 and p21 was in UVB group compared with controls. (**G**) Immunofluorescence showed that p53 and p21 were co-located in the nucleus of cells. *p<0.05.

SRA01/04 cells were exposed to UVB for 5 min, the number of SA-β-gal-positive cells increased than controls (Figure 3A). The results indicated that the senescence of cells was increasing. In addition, the *MRE11A* mRNA expression decreased during the prosses (Figure 3B), while the circMRE11A expression increased compared with controls (Figure 3C). The results were consistency with the expression in LECs of ARCC patients (Figure 1F). Meanwhile, CCK-8 showed that the cells viability decreased in UVB group compared with controls (Figure 3D). Moreover, Flow Cytometry demonstrated that cell cycle was inhibited in G1/S in the UVB group (Figure 3E). We further analysis the related proteins by Western Blot (Figure 3F). The immunofluorescence showed that p53 and p21 were co-located in the nucleus of cell lines (Figure 3G). The results showed that ATM expression was decreased in UVB group compared with controls. As the symbol of ATM activation, expression level of ATM-S1981p was increasing. Meanwhile, the expression of MRE11A decreased, and the relative increase of p53 and p21 was consistent with the previous results in LECs of ARCC (Figure 2B).

### Knock-down of circMRE11A enhances viability and cell cycle of SRA01/04 cell lines *in vitro*

**Figure 4:**
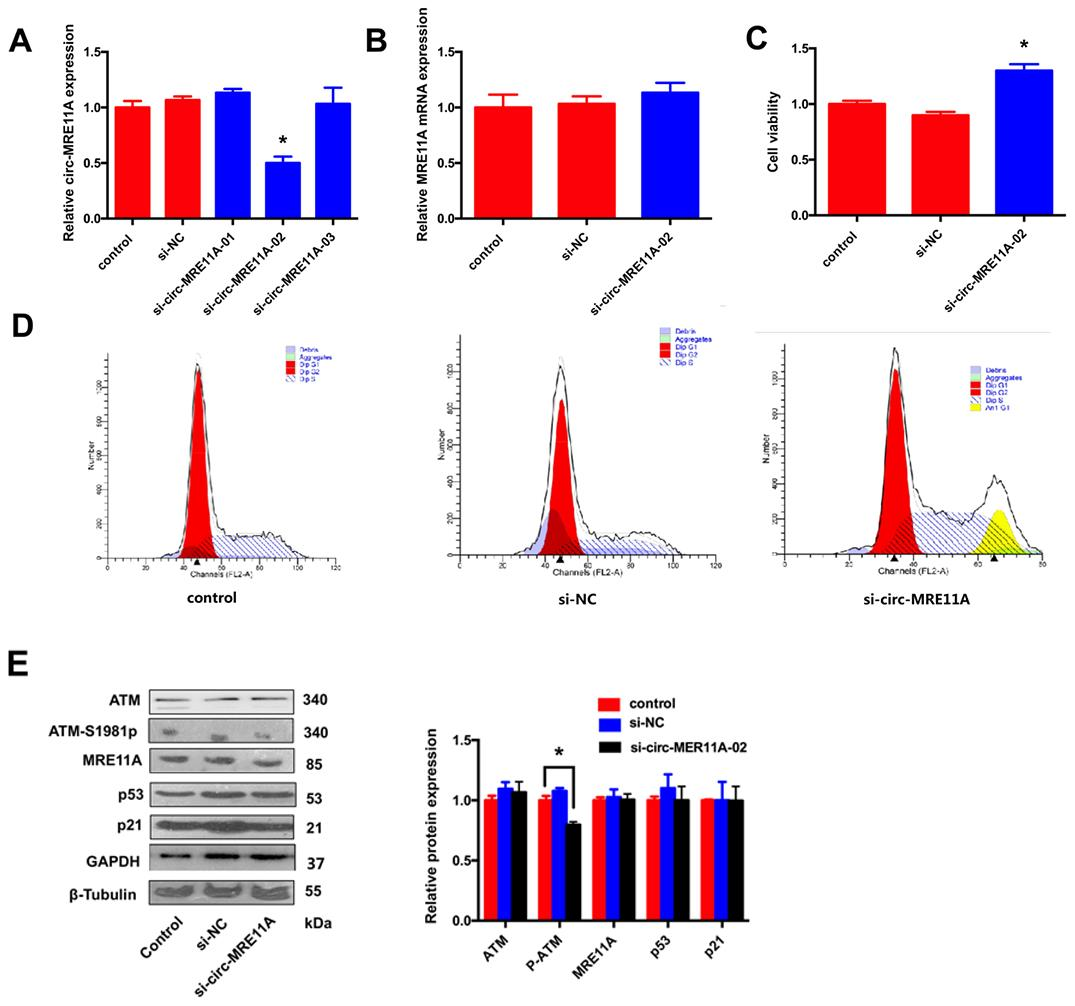
**Knock-down of circMRE11A enhanced viability of SRA01/04 cell lines *in vitro*.** (**A**). Designing three circMRE11A-specifific small interfering RNAs (siRNAs) targeted the back-splice junction sequence to knock down the expression levels of circMRE11A in LECs. Determining by qRT-PCR, the level of circMRE11A decreased after transfecting cells with siRNA-02 compared with the other siRNAs. *p<0.05. (**B**). SiRNA-02 targeting the back-splice junction of the circMRE11A which did not affect the expression of MRE11A mRNA. (**C**). CCK-8 showed that silencing of circMRE11A enhanced cell viability in the cell lines compared with controls. *p<0.05. (**D**). Flow cytometry demonstrated that silencing of circMRE11A promoted G1 entering S which means the progression of cells into the DNA synthesis phase (S phase). (**E**). Western blot showed that knockdown of circMRE11A deceased expression of ATM-S1981p, but not other proteins ATM, MRE11A, p53 and p21. Densities of bands were quantified by Image J software. Tubulin or GAPDH levels were measured in parallel, served as internal controls. *p<0.05.

In order to investigate the role of circMRE11A in LECs, three siRNAs were designed for knock-down of circMRE11A. The expression of circMRE11A decreased after transfecting cells with siRNA-02 (Figure 4A). Then, we found that siRNA-02 targeting the back-splice junction knocked down only the circMRE11A but not affect the expression of *MRE11A* mRNA (Figure 4B). Therefore, siRNA-02 was used in further research. In addition, CCK-8 showed that silencing of circMRE11A in the cell lines enhanced cell viability (Figure 4C). Moreover, Flow Cytometry demonstrated that silencing of circMRE11A promoted G1 entering S in the cell lines (Figure 4D). It is helpful in driving the progression of cells into the DNA synthesis phase (S phase). As shown in Western Blot (Figure 4E), the expression of ATM-S1981p was decreased but the expression of ATM, MRE11A, p53 and p21 were not inhibited when knockdown the circMRE11A. We inferred that knock-down of circMRE11A might down-regulate ATM activation (ATM-S1981p), which might have less control on cell cycle inhibition contrary to promote cell proliferation.

### Over-expression of circMRE11A inhibited cell viability and cell cycle of SRA01/04 cell lines *in vitro*

**Figure 5:**
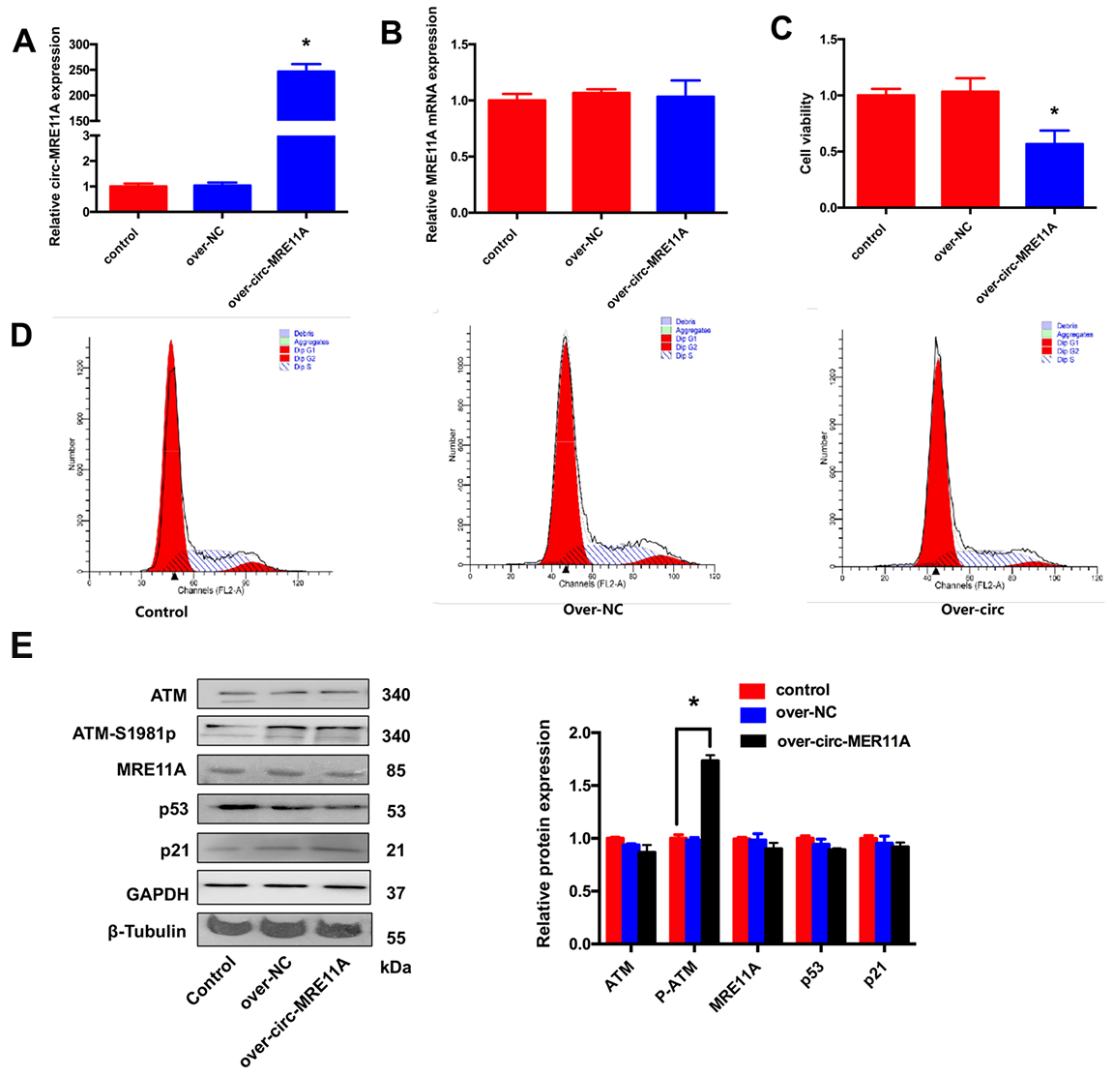
**Over-expression of circMRE11A inhibited cell viability and cell cycle of SRA01/04 cell lines *in vitro*.** (**A**). The qRT-PCR results showed that cells transfected with the construct expressed higher level of circMRE11A than that in the vector controls. *p <0.05. (**B**). Transfection with circMRE11A did not affect levels of MRE11A mRNA. (**C**). CCK-8 showed that the cell viability was decreased in the over-circMRE11A group compared with controls. *p <0.05. (**D**). Flow cytometry demonstrated that over-circMRE11A induced G1/S arrest in the cell lines. (**E**). The expression of ATM-S1981p was higher in the over-circMRE11A group compared with controls. Meanwhile, over-circMRE11A did not affect the levels of proteins MRE11A, ATM, p53 and p21. Tubulin and GAPDH levels were measured as internal controls. *p <0.05.

For further investigating the functional role of circMRE11A *in vitro*, whether circMRE11A is sufficient alone to affect cell cycle progress. We constructed plasmids which could over- expression circMRE11A or vectors as negative control (NC). We confirmed that the expression of circMRE11A was higher in over-circMRE11A group than the NC group (Figure 5A) while the expression of *MRE11A* mRNA was not changed (Figure 5B). Meanwhile, over-circMRE11A decreased cell viability (Figure 5C). Flow Cytometry demonstrated that over-circMRE11A induced G1/S arrest in the cell lines (Figure 5D). CircMRE11A transfection did not affect the expression of MRE11A or ATM (Figure 5E). However, the expression of ATM-S1981p was mildly higher in the over-circMRE11A group compared with NC group. CircMRE11A might enhance expression of ATM-S1981p and excessive activation of ATM might initiate cell cycle restraining and reduce cell viability. The results indicated up-regulation of circMRE11A may be involved cell cycle control though the modification of ATM proteins.

### CircMRE11A binds to UBXN1 may integrate ATM activation (ATM-S1981p)

**Figure 6:**
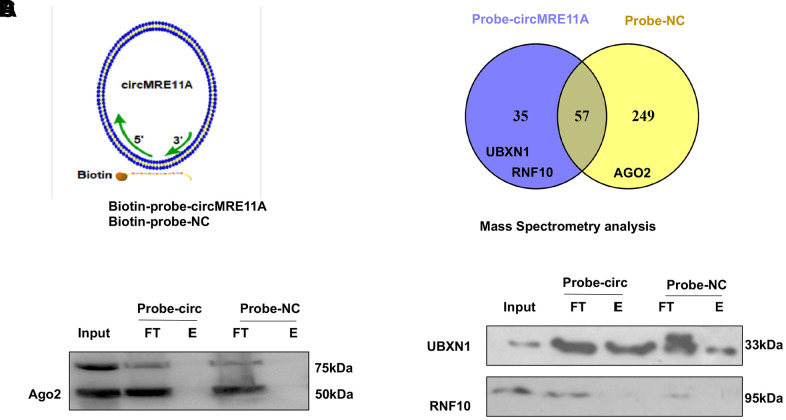
**CircMRE11A might bind to UBXN1 but not AGO2.** (**A**). The Biotin-probes using in RNA pull-down were to target the back-splicing junction region of the circMRE11A. This experiment also designed with sufficient Biotin-probe as negative control (NC). (**B**). The MS analysis confirmed that there was no AGO2 protein in 35 unique proteins of the circMRE11A-probe group compared with the NC-probe group. (**C**). Western blot showed the AGO2 was not enabled to precipitate by the Biotin-circMRE11A probes from precipitated complexes. (**D**). We selected the UBXN1 and RNF10 in 35 unique proteins of circMRE11A group. The precipitated complexes were further confirmed by Western blot, only UBXN1 was determined at the higher level in the circMRE11A-probe group compared with controls, but not RNF10. The results were consistency with MS analysis. Input was total proteins as an internal control.

The protein complexes were pulled-down by the specific probe of CircMRE11A. Then, we analysis the associated precipitate complexes which may bind to the RNA [32]. Designing probe used in RNA pull down was the sequence element, which was unique to target the back-splicing junction region of circMRE11A. However, circRNAs could pull down more non-specific binding proteins in the experiments. The assay should be carefully designed with sufficient negative control (NC) probe as controls (Figure 6A). The mass spectrometry (MS) results confirmed that there were no AGO2 protein in 35 unique proteins of the circMRE11A-probe group compared with the NC-probe group (Figure 6B). Furthermore, the precipitated complexes were confirmed by Western Blot (Figure 6C), the results also showed AGO2 protein was not precipitated in the circMRE11A-probe group. We believed circMRE11A might not sponge with miRNA, not bind to AGO2 at least. On the other hand, we selected the UBXN1 and RNF10 for further research. They were the only two ubiquitin ligases in 35 unique proteins in precipitated complexes of the circMRE11A-probe group. Then, the precipitated complexes were confirmed by Western Blot (Figure 6D), the results showed that only UBXN1 was determined and at a higher level in the circMRE11A-probe group compared with the NC-probe group. We assumed that circMRE11A might not sponge to miRNA but UBXN1, which might induce modification of ATM proteins in LECs cell cycle arrest through ATM/p53/p21 signaling pathway.

### ATM or ATM-S1981p was co-localization with UBXN1 in the cytoplasm of SRA01/04 cell lines

**Figure 7:**
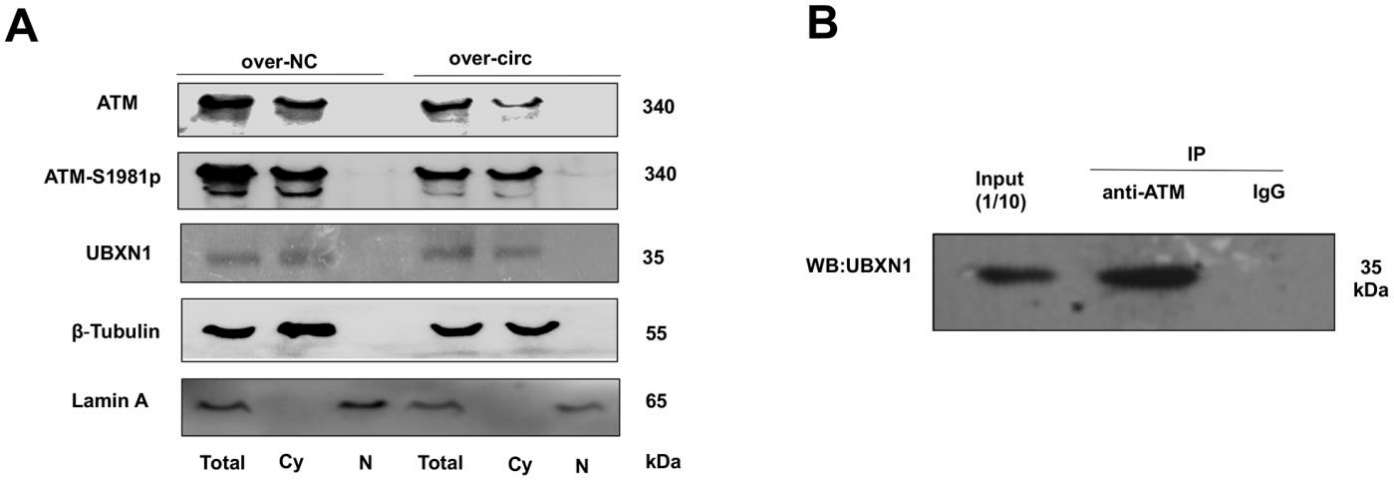
**ATM or ATM-S1981p was co-localized with UBXN1 in the cytoplasm of SRA01/04 cell lines.** (**A**). The cytoplasm and nuclei separation of proteins showed ATM, ATM-S1981p and UBXN1 were detected in cytoplasm from the over-circMRE11A group and the negative control (NC) group. We confirmed that increasing cytosolic fraction levels of ATM-S1981p but lower level of UBXN1 were detected in the over-circMRE11A group compared with the NC group. β-Tubulin as cytoplasm control, Lamin A as nuclear control, and total proteins as an internal control. (**B**). IP showed that UBXN1 could be precipitated by ATM in the cell lines. Input was 1/10 of total proteins as internal control.

For further investigating the possible relationship between circMRE11A, UBXN1 and ATM activation in LECs. CircMRE11A were determined in the cytoplasm of the cell lines (Figure 1D). Cytoplasm and nuclei separation of proteins assay showed that ATM and UBXN1 were co-located in the cytoplasm of the cell lines. β-Tubulin and Lamin A were used as the cytoplasm and nuclear proteins control. We isolated nuclear and cytosolic fractions of proteins in the cell lines and quantified cytoplasm ATM, ATM-S1981p and UBXN1 from the over-circMRE11A group and the negative control (NC) group. The results confirmed that increasing cytosolic levels of ATM-S1981p but low level of UBXN1 were detected in the over-circMRE11A group compared with the NC group (Figure 7A). Furthermore, Immunoprecipitation (IP) showed that UBXN1 could be precipitated by ATM in the cell lines, IgG as the negative control (Figure 7B). The results showed that UBXN1 might crosstalk with circMRE11A and combine with ATM in cytoplasm. The process might enhance excessive activation of ATM and initiate ATM/p53/p21 senescence signal pathway.

### *In vivo*, circMRE11A-AAV2 was constructed and intravitreal injection in ICR mouse

In current study, we found the infectious rAAV vectors which could pass through the capsule tissue of lens from the sizes of rAAV vectors. All the 7 virions of rAAV vectors were selected for the experiment. After intravitreal injection in ICR mouse at 4 weeks, the GFP- fluorescent protein encoded by rAAV vectors were detected under the fluorescence microscope. The results showed that rAAV-2 virions could express the highest level GFP-fluorescent in the lens of ICR mouse. Therefore, the infectious rAAV vectors rAAV-2 virions of circMRE11A (circMRE11A-AAV2) and negative control rAAV2 vector (NC-AAV2) were constructed for further research.

**Figure 8:**
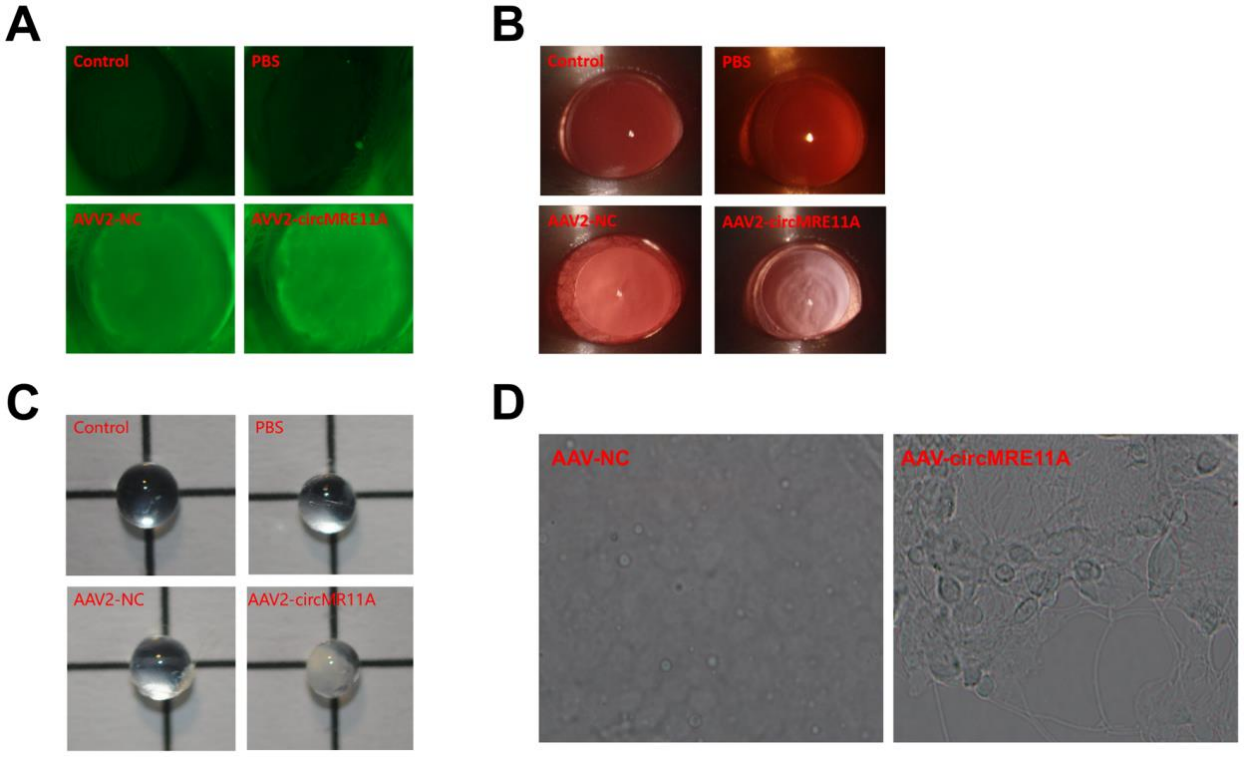
***In vivo*, circMRE11A-AAV2 was constructed and injected intravitreally in ICR mouse.** (**A**). After intravitreal injection at 4 weeks, GFP-fluorescent protein encoded by circMRE11A-AAV2 or NC-AAV2 were detected in the lens of the mice under the fluorescence microscope in the circMRE11A-AAV2 group or the NC-AAV2 group compared with the control group. The results showed that circMRE11A-AAV2 had passed through lens capsule tissue and expressed in the lens of mice. (**B**). After intravitreal injection at 8 weeks, the opacification of lens in the circMRE11A-AAV2 group was observed compared with the control group under the slit lamp microscope. (**C**). Taking out the eyeball of mice, and the lens was observed under light microscope. (**D**). The number of SA-β-gal-positive cells increased in circMRE11A-AAV2 group compared with control group.

After intravitreal injection in ICR mouse at 4 weeks, GFP-fluorescent proteins were detected from the lens of mouse under the fluorescence microscope in the circMRE11A-AAV2 group or the NC-AAV2 group compared with the control groups. The results showed that circMRE11A-AAV2 had passed through lens capsule and expressed in the lens (Figure 8A). Furthermore, the opacification of lens in the circMRE11A-AAV2 group was observed after intravenous injection at 8 weeks compared with the control groups under the slit lamp microscope (Figure 8B). Taking out the eyeball of mouse, and the lens was observed under light microscope (Figure 8C). The number of SA-β-gal-positive cells increased in circMRE11A-AAV2 group compared with control group (Figure 8D). It showed that circMRE11A-AAV2 could enter and express in the LECs of ICR mouse lens. The aging LECs and opacity lens of mouse were observed after intravitreal injection.

## DISCUSSION

ARC is a complex aging disorder involving environmental and genetic risk factors [5]. In recent years, some reports including our previous studies have shown UV radiation may be an important cataractogenic factor [4, 7, 33]. UVB irradiation efficiently induces DDBSs and LECs aging, which is an early event during cataract development especially in ARCC formation [34]. Consistent with previous study, p53 has been suggested to be closely involved in the aging pathogenesis of cataract [33]. Indeed, as the central controller of cellular responses to DNA damage, ATM controls the DNA repair network and preserve genome integrity [9, 35]. As inactive dimeric ATM protein dissociates into phosphorylated monomers. Persistent ATM activation leads to impaired DNA repair and cell survival [36]. However, excessive activation of ATM phosphorylates affects downstream molecule, including p53, contributes to multiple chronic diseases and age-related diseases [37]. Once DDR is completed, how and why ATM is not disassembled or deactivated remains unclear. For estimating clearly in early aging progress of LECs, we monitored excessive activation of the ATM by measuring ATM autophosphorylation at serine (S) 1981 (a site in the FAT domain, ATM-S1891p), which could occur at the activation of ATM in response to DNA damage [17]. The expression level of ATM protein was lower while ATM-S1891p is higher compared with controls under UVB-induced cell senescence (Figure 3F). Therefore, the excessive activation of ATM might extensively more induce LECs aging in ARCC.

In the regular pre-senescent process and age-related diseases progression onset, our investigation has revealed that DNA repair genes expression were altered under oxidative stress in ARCC [28]. But little known about the role of circRNAs derived from the DNA repair genes. Most circRNAs in human normal and aging tissues are low abundance and may be by-products in pre-mRNA splicing [38, 39]. Furthermore, certain circRNAs are predominately expressed in one gene locus and present at substantial levels [38]. However, circRNAs expression is cell type- and tissue-specific and can be largely independent of the expression level of the linear host gene. The majority of circRNAs are derived from precursor mRNA via exon circularization, indicating that a variety of additional transcripts can be produced from precursor mRNAs [40]. In this study, we characterized circMRE11A, one of the abundant circRNAs derived from Exon 6 and Exon 7 of the *MRE11A* gene (Figure 1A). In previous report, circMRE11A was also identified via the deep sequencing of several human cell lines [41]. Northern Blot and RT–qPCR confirmed that the formation of circMRE11A may be due to directly back-splicing of MRE11A mRNA. Thus, the regulation of expression might be an important aspect with regard to control of circular RNA function. But how and which may enhance the production of circMRE11A derived from Exon 6 and Exon 7 of the *MRE11A* gene need further research.

Accumulating evidence indicates that many circRNAs have been suggested to play a vital role during the regular aging process. In current study, we found that not MRE11A mRNA but circMRE11A significantly affected cell viability and cell cycle *in vitro*. CircMRE11A was up-regulated but MRE11A mRNA or proteins were low expression in both LECs of ARCC and the cell line treated with UVB. It suggests that circMRE11A serve a specific function in LECs aging-related progression. The growth-promoting function is carried out only by circMRE11A. Over-expression and knockdown of the circMRE11A had no influence on MRE11A mRNA expression, but appeared to be caused a critical functional on cell viability and cycle regulation. Obviously, circMRE11A might play a central role in LECs independent of the expression level of MRE11A mRNA or proteins.

Growing evidence have been reported that many circRNAs have functions as miRNA sponges, proteins scaffold as well as encode proteins with specific protein domains in numerous pathways and diseases [42, 43]. For further grasping the biological role of circMRE11A, the regulatory network might be considered in which the associated miRNAs. Notably, many studies mainly based on genome-wide bioinformatic analyses of all circRNA candidates and has less experimental validations. However, recent publication suggests that circRNAs do not necessarily function as miRNA sponges in human and mouse cells [23]. We also speculate that many circRNAs may not serve as miRNA sponges due to low abundance and small length. In another hand, some circRNAs could be sorted into exosomes and exported outside of the cells [24, 44]. In this study, RNA pull-down, MS analysis and IP showed that circMRE11A could serve as a modulator of cell growth by binding to UBXN1, but not AGO2 which could form silent compound with miRNAs (Figure 7). Therefore, the interaction and functional implication would be more common for circRNAs to associate with the scaffold of proteins [45].

Ubiquitin regulatory X (UBX) domain had been testified that it displayed weak amino acid sequence homology to ubiquitin. By far, many UBX domain containing proteins are revealed to be able to bind and act as co-factors of downstream protein, which could induce or improve PTM of proteins [46]. UBXN1 contains both N-terminal UBA domain and C-terminal UBX domain. It is a central component in many ubiquitin-mediated pathway and play pivotal roles in many cellular processes, including cell cycle and DNA damage [46, 47]. Moreover, it is considered primarily as the PTM to initiate proteasome mediated degradation via polyubiquitination. Protein ubiquitination represents a complex system for modifying protein function by changes in interactions of protein, subcellular localization, or enzyme activity [48]. However, it was unclear whether UBXN1 participates in the regulation of progression of ARC formation. As showed in (Figure 7), UBXN1 was detected and combined with circMRE11A and linked to ATM in cytoplasm. These results showed that interaction of UBXN1and circMRE11A might integrate ATM excessive activation, which might play a critical role in regulating of ATM/p53/p21 aging signaling pathway. However, the expand roles of UBXN1 in the regulation of ATM activation and the progression of ARCC formation need to be further estimated.

In current research, the rAAV2 vectors was selected from the 7 virions of rAVV vectors, which may pass through the capsule tissue of lens and express the highest level GFP-fluorescent in the lens of ICR mouse after intravitreal injection at 4 weeks. Human *MRE11A* gene is highly conserved to mouse *mre11a*. The sequence in the junction area of circMRE11A is different from the other species. However, circMRE11A has 63% homology with the mouse genome. For further research circMRE11A *in vivo*, circMRE11A-AAV2 was constructed. As showed in Figure 8A, 8B, circMRE11A-AVV2 were detected and expressed in lens of ICR mouse after intravitreal injection. Furthermore, the opacification of lens was observed and cell aging is increasing after intravitreal injection at 8 weeks (Figure 8B). Although the results could not estimate clearly. We provide a new vector (rAAV2) that could pass through and enter into the lens tissue.

Our study characterized and functionally evaluated an abundant circMRE11A derived from the Exon 6 and Exon 7 of the MRE11A gene. The identification of circMRE11A expanded our understanding of the complex regulation of DNA repair gene in ARC formation under UVB-induced oxidative stress. Moreover, ATM was the heart of DNA repair network, but excessive activation of the ATM might eventually induce LECs aging in the prosses. As the scaffold of UBXN1, our findings suggested that circMRE11A might take part in the progress of LECs cycle control through integrating ATM excessive activation, enhancing cell cycle inhabitation in ARCC formation. *In vivo*, after intravitreal injection in ICR mouse, circMRE11A-AAV2 could pass through the capsule tissues of lens and express in the lens. Those results can provide a better understanding of ARC pathology as well as novel and more effective therapeutic target.

## MATERIALS AND METHODS

### Study participants and human tissue samples

This study was approved by the Ethics Committee of the Affiliated Hospital of Nantong University and was conducted in adherence to the tenets of the Declaration of Helsinki. All participants were explained the purpose and procedures of the study and signed informed consent. All lens samples were obtained at the Affiliated Hospital of Nantong University from November 2018 to January 2019. The lens samples were obtained by intact continuous curvilinear capsulorhexis, without vascular contacting or damage to the iris or any other intraocular tissues. The degree of lenticular opacification was determined according to the Lens Opacities Classification III (LOCS III) classification [49]. Age-matched individuals who had their transparent lens extracted because of vitreoretinal diseases were included as controls. Patients with complicated cataracts due to high myopia, trauma, uveitis, and glaucoma or with systematic diseases, such as hypertension and diabetes were excluded from the study. All samples were divided into two parts. One part was rapidly mixed Trizol reagent (Invitrogen, Thermo Fisher Scientific, Waltham, MA, USA) and then stored at -80° C until RNA extraction. The other part was stored at -80° C until protein extraction. The basic demographic of the study participant was listed in the Table 1.

**Table 1:** The grade of lens opacity and identification codes of controls and ARCs.

**Controls**				**ARC-C**				**ARC-N**				**ARC-P**			
**Samples**	**Sex**	**Age(y)**	**LOCSIII**	**Samples**	**Sex**	**Age(y)**	**LOCSIII**	**Samples**	**Sex**	**Age(y)**	**LOCSIII**	**Samples**	**Sex**	**Age(y)**	**LOCSIII**
No.1	Male	60	C1N0P0	No.1	Male	75	C3N1P0	No.1	Male	78	C1N3P2	No.1	Male	75	C1N1P2
No.2	Male	65	CON1P0	No.2	Male	68	C3N2P0	No.2	Male	68	C1N3P0	No.2	Male	62	C1N0P3
No.3	Male	55	C0N1P0	No.3	Male	71	C3N3P0	No.3	Male	63	C1N3P0	No.3	Male	71	C1N0P3
No.4	Male	60	CON IPO	No.4	Male	75	C3N1P0	No.4	Male	69	C1N3P0	No.4	Male	71	C1N0P3
No.5	Male	55	C0N1P0	No.5	Male	63	C3N1P0	No.5	Male	64	C1N3P0	No.5	Female	63	C1N0P3
No.6	Female	68	C1N1P0	No.6	Female	63	C2N0P0	No.6	Female	70	C0N3P0	No.6	Female	72	C1N0P3
No.7	Female	65	C0N1P0	No.7	Female	75	C3N1P0	No.7	Female	72	C0N3P0	No.7	Female	63	C0N1P3
No.8	Female	65	C0N2P0	No.8	Female	72	C3N1P0	No.8	Female	70	C0N3P0	No.8	Female	58	C1N0P3
No.9	Female	59	CON2PO	No.9	Female	65	C3N1P0	No.9	Female	69	C0N3P0	No.9	Female	63	C1N0P3
No.10	Female	54	CON1P0	No.10	Female	61	C0N3P0	No.10	Female	61	C0N3P0	No.10	Female	61	C0N1P3

### RNA extraction

Total RNAs were extracted using Trizol reagent (Invitrogen) according to our previous study [50]. Cytoplasmic and nuclear RNA and proteins of SRA01/04 cell line were isolated by PARIS™ Kit Protein and RNA Isolation System (Invitrogen) according to the manufacturer’s instructions. The yield of RNA was detected using a NanoDrop 2000 spectrophotometer (Thermo Fisher Scientific, Inc, USA).

### RNA treatment with RNase R, sanger sequencing and Reverse transcription-quantitative PCR (qRT-PCR)

For Ribonuclease R (RNase R, E. coli, Epicentre Technology, Madison, WI, USA) treatment, 2 μg total RNA was incubated for 30 minutes(min) at 37° C with or without 2 U/μg RNase R. Then the enzyme was inactivated at 85° C for 5 min and the product was purified by RNeasy mini kit (QIAGEN, GmBH, Germany). To validate the existence of a circRNA, Sanger sequencing was performed by GENEWIZ services (Sangon Biotech, Shanghai, China) for direct PCR product amplified with the divergent primers.

qRT-PCR was performed by using SYBR Green (Applied biosystems, Thermo Fisher Scientific, USA) according to the manufacturer’s instructions to validate differentially expressed circRNAs or mRNAs. Quantification was performed with a two-step reaction process RT and qRT-PCR. Each RT reaction consisted of 1 μL RNA, 1 μL RiboLock RNase inhibitor (40 U/μL) (Invitrogen), 1 μL dNTP mix (10 mM each) (Invitrogen), 1 μL random primers (200 ng/μL) (Sigma-Aldrich, Roche Diagnostics, USA) and 1 μL Maxima Reverse Transcriptase (Invitrogen), then added to a total volume of 20 μL with RNase Free dH_2_O (Invitrogen). Reactions were performed using a PCR system 7500 (Applied Biosystems) for step 1, 65° C for 30 min, followed by heat inactivation of RT for 5 min at 85° C. The 20 μL RT reaction mix was then diluted 10-fold with nuclease-free water (Invitrogen) or held at -80° C. RT-qPCR was performed by using SYBR Green (Applied Biosystems) according to the manufacturer's instructions in ABI 7500 system (Applied Biosystems). PCR reaction mixture included 5 μL cDNA, 10 μL 2 × greenStar Master mix, 2 μL divergent primers (1 μL forward primer, 1 μL reverse primer,10 nM each) and added to a total volume of 20 μL with PCR grade water. The thermocycling conditions were as follows: 95° C for 10 min followed by 40 cycles of 95° C for 10 s, 60° C for 30 s. PCR products were checked on 2% agarose gel. Primer sequences were listed in Supplementary Table 1. To normalize the data, the average expression of GADPH, data quantification was carried out using the 2^−ΔΔCT^ method.

### Northern blot

Northern Blot were performed with NorthernMax^®^ Kit (Ambion, Thermo Fisher Scientific, USA) following manufacturer’s instructions. After RNase R (E. coli Epicentre) treatment, about 20 μg of total RNA was incubated for 10 min or 30 min at 37° C with or without 2 U/μg RNase R and enzyme was inactivated at 85° C for 5 min. Then the RNAs were run on 1% agarose gel and transferred to a Hybond-N+ membrane (GE Healthcare, Thermo Fisher Scientific, USA). The membranes were dried at 85° C for 30 min and then ultraviolet-crosslinked (at 265 nm) for 15 min. Pre-hybridization was first conducted at 68° C for 2 h. Hybridization was then conducted at 68° C overnight. The membranes were visualized through North2South^®^ Chemiluminescent Hybridization and Detection Kit (Pierce Biotechnology, Rockford, IL, USA) following manufacturer’s instructions. The sequence of bioth-probes for circMRE11A and 18s RNA were listed in Supplementary Table 2.

### Fluorescence *in situ* hybridization (FISH)

Briefly, Cy3-labeled circMRE11A probe were designed and synthesized by Geneseed Co., Ltd. (Guangzhou, China). The probe signals were detected with a fluorescent *in situ* hybridization kit (RiboBio, Guangzhou, China) according to the manufacturer’s instructions. The labeled probe flanking circMRE11A was applied to cells. The cells were treated with 4’-6-diamidino-2-phenylindole (DAPI; SouthernBiotech, Birmingham, USA) as a nuclear control. The results were observed under a fluorescence microscope (Eclipse E600; Nikon Corporation, Tokyo, Japan).

### Senescence-associated β-galactosidase staining

β-gal staining was performed using senescence-galactosidase staining kit (Beyotime Biotechnology, Shanghai, China) according to the manufacturer’s instructions. In brief, cells were cultured in 10% FBS/DMEM to sub-confluence and then fixed in 3.7% formaldehyde for 5 min, washed in Hankes’ Balanced Salt Solution (HBSS; Beyotime Biotechnology). The treatment or control cells were stained at 37° C until staining become visible. The number of positive cells were subsequently observed and counted under a light microscope (Leica, Germany).

### Immunofluorescence assay

LECs were fixed with 4% paraformaldehyde for 10 min, and then permeabilized with 0.5% Triton X-100 for 15 min. The cells were washed with HBSS for three times, nonspecific binding sites were blocked with 3% bovine serum albumin for 2 h. These cells were incubated with the primary antibody overnight at 4° C, and then incubated with the secondary antibody conjugated with Alexa Fluor 488 or 568 (Invitrogen) for 3-4 h at room temperature, followed by incubation with DAPI (Southern Biotech) for 5 min. Immunofluorescence was observed under the fluorescence microscope (Leica, Germany).

### Cell culture and UVB irradiation

Human LEC line (SRA01/04 cell, Riken, Japan) was cultured in Dulbecco’s modified Eagle’s medium (DMEM, Invitrogen-GIBCO, Carlsbad, CA, USA) with 10% heat-inactivated fetal bovine serum (FBS, GIBCO, Carlsbad, CA) and 100 U/mL penicillin, in a humidified atmosphere containing 5% CO_2_ at 37° C. Unless otherwise indicated, the cells were passaged at 70%-80% confluency, then serum-starved overnight prior to treatment, and all experiment used cells within thirdly generations of the original cells. UVB irradiation treatment was described in our previous study [50]. At 24 h after UVB exposure, total RNAs were extracted from the cells with Trizol reagent (Invitrogen). The cells were also used for other analysis such as CCK-8 and cell cycle analysis. Then, cDNA was synthesized as previous recommended. Total proteins were collected from the cells at 48 h after UVB exposure.

### CCK-8 assay

Cell viability was detected by Cell Counting Kit 8 assay (Dojindo Laboratory, Kumamoto, Japan). After specific treatment of the cells, 10 μL of CCK-8 was added to each well of a 96-well plate, and continually incubated at 37° C for 1-2 h. The absorbance at 405-450 nm wavelength was detected using a microplate reader (BioTek, VT, USA). Then, the cell viability was calculated.

### Propidium iodide staining and flow cytometry (FCM)

Propidium iodide (PI) staining (BD, Franklin Lakes, NJ) was used to quantify cell cycle analysis. SRA01/04 cells were fixed with 75% ethanol for overnight at -20° C before stained with PI. After washing with HBSS for three times, the stained cells were analyzed by a FACS Calibur Flow cytometer (BD, NJ, USA).

### siRNA transfection

For knock-down of circMRE11A, SRA01/04 cells were transfected with siRNAs using Lipofectamine 3000 (Invitrogen). In brief, 4×10^4^ cells in 2 mL of DMEM (10% FBS) were plated in 6-well plates and incubated overnight at 37° C and 5% CO_2_ atmosphere. The cells were cultured in serum-free medium 1h before transfection. For each dish, 2.5 μL siRNA was added into 150 μL of serum-free medium and then added into another 150 μL of serum-free medium mixed with 2.5 μL of Lipofectamine 3000. The mixture was added to the cells and incubated for 6 h before replacing the medium with DMEM containing 10% FBS. The cells were harvested for function assay or RNA extraction 24 h later. For proteins preparation, cell lysates were prepared 48 h after transfection. The sequences of siRNA for circMRE11A were listed in Supplementary Table 3.

### Plasmid construction and transfection of circMRE11A

The over-expression vector of circMRE11A (pK25ssAAV-ciR-hsa_circ_000237) and control (pK25ssAAV-ciR) were constructed and generated by Geneseed Co., Ltd. Both plasmids contain a Bluescript backbone, a CMV promoter driving green fluorescent protein (GFP) expression and a human H1 promoter driving circMRE11A. For over-circMRE11A, cells were transfected with plasmids using Lipofectamine 3000 (Invitrogen). In brief, 4×10^4^ cells in 2 mL of DMEM (10%FBS) were plated in 6-well plates and incubated overnight at 37° C and 5% CO_2_ atmosphere. The cells were cultured in serum-free medium 1 h before transfection. For each dish, 2.5 μg plasmids was added into 150 μL of serum-free medium mixed with 2.5 μL of p3000, and then added into another 150 μL of serum-free medium mixed with 2.5 μL of Lipofectamine. The mixture was added to the cells and incubated for 6 h before replacing the medium with DMEM containing 10% FBS. Cells were harvested for function assay or RNA extraction 24 h later. For proteins analysis, cell lysates were prepared 48 h after transfection.

### RNA pull-down assays and mass spectrometry (MS) analyses

RNA pull-down assays were performed using the Pierce™ Magnetic RNA-Protein Pull-Down Kit (Thermo Fisher) according to the manufacturer’s instruction. The pull-down assay was performed using RNA probes complementary to circMRE11A. The probes were designed and labelled with biotin. The selected probes were confirmed not to recognize nucleotide sequences in other RNA expressed in the chosen cells [32]. Briefly, after over-circMRE11A in the cells at 24 h, 1×10^7^ cells were washed in ice-cold HBSS, lysed in 1 mL IP buffer (Invitrogen) for further study. Washing 100 μL Streptavidin C1 magnetic beads (Invitrogen) with yeast RNA (Ambion, Life Technology, Carlsbad, CA, USA) or NaCl (100 uM) were added to each binding reaction and further incubated with 3 μg biotinylated circMRE11A oligo probes or negative control (NC) probes. The streptavidin-linked magnetic beads were incubated with total over-circMRE11A group cell lysates at 4° C for 2 h. The bead-RNA-protein complexes were washed with 1× binding washing buffer three times. The proteins were precipitated and diluted in protein lysis buffer. Finally, the retrieved proteins were measured for one-shot mass spectrometry (MS) in Genechem Co., Ltd. (Shanghai, China) or Western blot. RNA pull-down specificity was assessed by Western blotting with samples normalized by volume and bands. Detailed information regarding the probes is depicted in Supplementary Table 2.

### Immunoprecipitation (IP)

IP assays were performed using the Pierce™ Direct Magnetic IP/Co-IP Kit (Thermo Fisher) according to the manufacturer’s instructions. The cells were washed with ice-cold HBSS, and lysed in 1mL lysis buffer (Invitrogen). Washing equal 50 μL Magnetic Beads (A/G) (Invitrogen) with yeast RNA (Ambion) or IP Lysis/Wash Buffer, the cells were further incubated with 5 μg primary antibody or IgG as negative control (NC) for 1 h. Equal amounts of proteins were incubated with the antibody-coupled magnetic beads for 4 h at 4° C on a rotator. The pellets were washed 3 times with lysis buffer (Invitrogen) and resuspended in Elution Buffer (Invitrogen), followed by Western Blot analysis.

### Western blot assays

SRA01/04 cells were collected and lysed in RIPA containing phenylmethanesulphonyl fluoride (Beytime, Shanghai, China) at 4° C for 30 min. Protein concentrations of the cells extraction were measured by a BCA Protein Assay Kit (Thermo Scientific). The proteins were size fractionated by sodium dodecyl sulfate-polyacrylamide gel electrophoresis on 5-12% polyacrylamide gels and transferred onto polyvinylidene difluoride membranes (Millipore, Bedford, MA, USA). Nonspecific protein binding to the membranes was blocked with blocking buffer (5% nonfat milk, 200 mM NaCl, 50 mM Tris, 0.05% Tween 20). The blocked membrane was then incubated with the primary antibody (Supplementary Table 4) at 4° C for 12 h. The membranes were incubated with the secondary antibody for 2 h after washed three times with TBS-T (20 mM Tris, 500 mM NaCl, 0.1% Tween 20) for 5 min each time. After washing in phosphate-buffered saline (PBS) for three times, the membranes were exposed to light film (BioMax MR; Kodak, Rochester, NY, USA) using an ECL Kit (Pierce, Rockford, IL, USA).

### Animals and surgery

All animal experiments were approved by the Animal Care Committee of Nantong University (approval number: Y20080290), and undertaken according to NIH Guidelines for the Care and Use of Laboratory Animals. Adult ICR mouse with body weight of 20 and 30 g of two months were obtained from the animal facility of Nantong University. Animal experiments reported in this study were conducted with ICR mouse bred and maintained in accordance to NIH guidelines. *In vivo*, circMRE11-AAV2 or AAV2-NC (1×10^12^) were subcutaneously injected into the eyeball vitreous cavity of blindly randomized ICR mouse eye as previous described [51] (n = 5 per group). Prior to injection, the pupils of mouse were dilated with 2.5% phenylephrine HCl (Akorn, Lake Forest, IL, USA) ophthalmic solution. The mice were anesthetized using 200 mg/kg pentobarbital sodium. A small hole was made at the limbus of the eye with a 30 G needle, then 0.5 μL virus (at a dose of 1x10^12^ vector genomes) was slowly delivered with a 34 G needle on a Hamilton gastight syringe. All mice were killed following deep anesthesia with pentobarbital sodium after the injection at 8 weeks and their lenses were extracted carefully.

### Statistical analysis

The one-way analysis of variance (ANOVA) test was performed to identify the differences among the groups. Differences were considered significant when the *P* <0.05. Statistical analysis was performed by SPSS software (SPSS 20.0; SPSS, Inc., USA).

## Supplementary Material

Supplementary Tables
