# Supplementary material for: CircMRE11A_013 binds to UBXN1 and integrates ATM activation enhancing lens epithelial cells senescence in age-related cataract
Source: Aging (Albany NY). 2021 Jan 28;13(4):5383–402. doi: 10.18632/aging.202470 (PMC7950295; doi:10.18632/aging.202470)
Supplement: Supplementary Tables [file aging-13-202470-s001.pdf]

SUPPLEMENTARY TABLES

Supplementary Table 1. The primers of RT-PCR.

Gene	Primer (5'-3’)
circMRE11A-F	CCCGTAGCAGGAACTCAAAG
circMRE11A-R	CAGCACTAAAGGCAGAAGCA
MRE11A F	CTTGTACGACTGCGAGTGGA
MRE11A-R	TTCACCCATCCCTCTTTCTG
ATM-F	AGGCTGGAGTGTGGTGGTGAG
ATM-R	TGTGATGGCTTGTGTCTGTGGTC
p53-F	ATGGAGGAGCCGCAGTCAGAT
p53-R	GCAGCGCCTCACAACCTCCGTC
p21-F	TGTCCGTCAGAACCCATGC
p21-R	AAAGTCGAAGTTCCATCGCTC

Supplementary Table 2. The sequence of bioth-probes.

Probe	Sequence (5'-3')
Bioth-probe-circMRE11A	TCTGGCCCTGGTCATAGCCATCTATAATAGACATATTTCT
Bioth-probe-NC	TCTGGCCCTGGTCATAGCCGCCTGTAATCCCAGCACTTTG
Bioth-probe-18SRNA	TTACCGCGGCTGCTGGCACCAGACTTGCCCTCCAATGGATCCTCGTTAAAGGATTTAAAGTGGA

Supplementary Table 3. The primers of overexpressed vectors and siRNAs.

Gene	Primer (5'-3')
h-circMRE11A-F	AGCATCGAGAGGAGGGTCTC
h-circMRE11A-R	AGTCATTAGCCATCTGTTC
h-GAPDH-F	GCTGAGAACGGGAAGCTTGT
h-GAPDH-R	GACTCCACGACGTACTCAGC
siRNA-NC	TTCTCCGAACGTGTCACGT
si-h-circMRE11A_01	ATTATAGATGGCTATGACC
si-h-circMRE11A_02	TATAGATGGCTATGACCAG
si-h-circMRE11A_03	TAGATGGCTATGACCAGGG

Supplementary Table 4. List of antibodies.

Used	Antibodies	Source and indentifier	Concentration (dilution)
Western	Mouse-Anti-ATM	GTX70103 (2C1), GeneTex	1/2000
Blot	Anti-ATM (phospho S1981)	Abcam, ab81292,	1/10000
	Mouse-MRE11	GeneTex, 12D7, GTX70212	1/2000
	Rabbit-Anti-p53	Abcam, ab131442	1/1000
	Rabbit-Anti-p21	Abcam, ab109520	1/5000
	Rabbit-Anti-GADPH	Abclonal, AC001	1/10000
	Rabbit-Anti-RNF10	Abcam, ab160716	1/2000
	Rabbit-Anti-UBXN1	Abcam, ab151723	1/2000
	Rabbit-Anti-Argonaute-2 antibody	Abcam, ab32381	1/2000
	Mouse-Anti-α-Tubulin	Sigma, T6199, CB84669834	1/10000
IF	Rabbit-Anti-p21	Abcam, ab109520	1/250
	Mouse-Anti-p53	Abcam, ab1101	1/200
IP	Rabbit-Anti-ATM	Abcam, ab199726	5ug/ml
	Mouse-Anti-IgG	Santa cruz, SC-2025	5ug/ml
